# Compensatory increase in oxygen extraction fraction is associated with age-related cerebrovascular disease

**DOI:** 10.1016/j.nicl.2025.103746

**Published:** 2025-01-28

**Authors:** John McFadden, Julian Matthews, Lauren Scott, Karl Herholz, Ben Dickie, Hamied Haroon, Oliver Sparasci, Saadat Ahmed, Natalia Kyrtata, Geoffrey J.M. Parker, Hedley C.A. Emsley, Joel Handley, Maélène Lohezic, Laura M. Parkes

**Affiliations:** a Division of Psychology, Communication and Human Neuroscience, School of Health Sciences Faculty of Biology, Medicine, and Health the University of Manchester UK; b Division of Informatics, Imaging and Data Science, School of Health Sciences Manchester UK; c University Hospital of Morecambe Bay NHS Foundation Trust Lancaster UK; d Bioxydyn Limited Manchester UK; e Centre for Medical Image Computing Department of Medical Physics & Biomedical Engineering and Department of Neuroinflammation University College London London UK; f Lancaster Medical School Lancaster University Lancaster UK; g Department of Neurology Lancashire Teaching Hospitals NHS Foundation Trust Preston UK; h Applications & Workflow GE Healthcare Manchester UK; i Geoffrey Jefferson Brain Research Centre Manchester Academic Health Science Centre Manchester UK

**Keywords:** OEF, QSM, CBF, Cerebrovascular disease, Cognitive impairment, Multi-PLD ASL

## Abstract

Cerebrovascular disease is an important contributor to dementia with reductions in cerebral blood flow (CBF) potentially compromising oxygen supply. In early stages, reduced CBF may be associated with a compensatory increase in oxygen extraction fraction (OEF) to maintain the cerebral metabolic rate of oxygen consumption (CMRO_2_). We used a simultaneous PET-MRI protocol to measure OEF, CBF, CMRO_2_, and arterial transit time (ATT) in elderly people (n = 24, age 69.6 ± 5.3 years) with a range of vascular disease risk (QRisk 18.7 ± 10.8 %) and cognitive abilities (MoCA scores 26.7 ± 3.4) to determine if a) vascular disease risk (parameterised with QRisk2 score) is associated with altered CBF, ATT, OEF and CMRO_2_, b) if impaired blood supply and increasing transit times are associated with elevated OEF and c) if these physiological measures are associated with impaired cognition. ATT rose by 132 ms per 10 point increase in QRisk and there was a trend for reduced CBF. Compensatory increases in OEF occurred in association with modified ATT and CBF, preserving CMRO_2_. There was no regional variation to these relationships. Cognitive impairment was associated with prolonged ATT. These findings demonstrate the potential use of multi-delay time ASL and Quantitative Susceptibility Mapping for the early detection of cerebrovascular changes and provide evidence for compensatory increases in oxygen extraction in the presence of reduced blood flow.

## Highlights

• Regional OEF, ATT, CBF, and CMRO2 were estimated in elderly people using MRI.
• ATT was longer in those with more vascular risk and worse cognition.
• OEF was elevated in those with longer ATT and lower CBF.

## 1 Introduction

Chronic cerebrovascular disease can lead to debilitating deterioration in an individual’s quality of life through vascular cognitive impairment (van der Flier, 2018, Dichgans and Leys, 2017), or as a comorbidity potentially accelerating conditions such as Alzheimer’s disease or Parkinson’s disease (Al-Bachari et al., 2017). This is apparent from the greater abundance of vascular disease in people with dementia, including degradation of the blood brain barrier (Popescu, 2009, Raja et al., 2018); reductions in the contractile ability of capillary pericytes (Nortley, 2019); an overall decrease in microvascular density; increasing abundance of string vessels; increased vascular tortuosity, coiling, and glomerular loops (Buée, 1994). The effect on cognition may be distinct from or enhanced by that caused by the formation of amyloid-β plaques, which has been well established as a marker of cognitive decline (Hanseeuw, 2019). However, understanding of how, and to what extent, vascular pathology, beyond what is seen in normal aging (Leidhin, 2021), provokes cognitive degeneration is lacking. We attempt in this work to develop this understanding by determining if vascular dysfunction detected in elderly individuals with a range of vascular risk scores and cognitive impairment conforms with a known model where reduced oxygen supply is associated with increased oxygen extraction, and to what extent various markers of vascular dysfunction are associated with cognitive impairment. We investigated vascular dysfunction using MRI data collected during a PET-MR study which examined the utility of a variety of novel MRI imaging methods, with amyloid PET data also being collected in order to aid the classification of patients as having the early stages of vascular dementia only, mixed dementia, or prodormal Alzheimer’s disease.

Cerebral blood flow (CBF) is a key parameter in mechanistic models of vascular supply to the brain, describing the blood volume that flows through a given mass of brain tissue per unit time (Fantini et al., 2016), and has been shown to be altered in dementia and vascular disease (Asllani, 2008). It has also been shown to decrease in grey matter during healthy aging (Leidhin, 2021, MacDonald, 2020, Alisch, 2021). Oxygen is a critical molecule exchanged between the blood and tissue, the degree of which can be quantified by the oxygen extraction fraction (OEF), defined as (Yablonskiy et al., 2013):

$$\mathrm{OEF}=\frac{{\mathrm{SaO}}_{2}-{\mathrm{SvO}}_{2}}{{\mathrm{SaO}}_{2}}$$

where SaO_2_ is oxygen saturation within arterial blood and SvO_2_ is the oxygen saturation within venous blood. This definition does not take into account the smaller contribution of dissolved oxygen, which is negligible in veins. CBF and OEF together can be used to estimate the cerebral metabolic rate of oxygen consumption (CMRO_2_ in μmol O_2_/min/100 ml tissue):

$${\mathrm{CMRO}}_{2}=CBF∙{\left[O_{2}\right]}_{a}∙OEF$$

where [O_2_]_a_ is the concentration of oxygen within arterial blood (in μmol O_2_/ 100 ml blood). If baseline metabolic activity cannot be maintained, this may disrupt cognitive function. Therefore, measurements of CBF, OEF, and CMRO_2_ may have utility in identifying disruption in oxygen supply associated with vascular disease.

Arterial spin labelling (ASL) is a non-invasive MRI method to measure CBF. A more accurate estimation of CBF with this technique uses the simultaneous measurement of the arterial transit time (ATT, the time required for blood to travel from the labelling slab to the capillary bed), which can be achieved using multi-delay time ASL (Woods et al., 2024). ATT is also a measure of interest in its own right as it could be associated with cerebrovascular resistance to blood flow on the arterial side, with increased resistance reducing flow velocity, and so prolonging ATT. In fluid dynamics, this resistance is a product of the length and diameter of a blood vessel and so is reflective of the underlying anatomy of the capillary network, with fewer, longer or narrower vessels leading to greater resistance and prolonged ATT. ATT is dominated mainly by transit through the arterioles and capillaries where velocity is lowest. Vessel diameters and blood flow rates, and therefore ATT and CBF, of feeder vessels are, in turn, products of small vessel haemodynamics (Gould et al., 2017). According to models of oxygen exchange such as the Krogh model (Krogh, 1919), alterations in the capillary network such as tortuosity, length and density, may impede oxygen extraction and manifest as altered OEF.

An effective MRI measurement of regional OEF has, until recently, proved challenging. Techniques such as dual-calibrated functional MRI (fMRI) (Bulte, 2012, Gauthier and Hoge, 2013, Wise et al., 2013) and quantitative blood-oxygen-level-dependent (BOLD) (He and Yablonskiy, 2007) imaging provide maps of OEF but require long acquisition times and/or use of physiological assumptions in the modelling that may not hold in disease. Susceptibility based oximetry (SBO) (Wehrli et al., 2017) exchanges regional information for a rapid estimation of global OEF, but still relies on assumptions on vessel geometry. T_2_-relaxation-under-spin-tagging (TRUST) (Liu, 2016, Jiang, 2018, Liu et al., 2013) is limited to a global measurement. Here, we use quantitative susceptibility mapping (QSM), a technique which has been shown to provide coarse regional estimates of OEF, with the resolution at acquisition and subsequent minimum resolvable vessel diameter as the determinant of coarseness of regional specificity (McFadden, 2021).

Our first aim therefore is to investigate any association between CBF, OEF, ATT, and CMRO_2_ and subclinical cerebrovascular disease, parameterised by cardiovascular disease risk (QRisk2). We hypothesize that CBF will be reduced in subclinical cerebrovascular disease and ATT prolonged. We examine this on a regional basis as we hypothesised that frontal and parietal regions may be more affected by vascular disease (Schuff, 2009). Second, we seek to investigate if there is a compensatory relationship between OEF and both CBF and ATT, predicting that OEF will be elevated to compensate for reduced CBF to maintain CMRO_2_ and will also be associated with prolonged ATT. Positive findings would support testing the hypothesis that, where compensation is insufficient, a reduction in CMRO_2_ associated with elevated QRisk would occur. Finally, we consider whether our physiological measurements are associated with cognitive impairment, above and beyond the impact of amyloid. We include test re-test data from a subset of our cohort and calculate inter-session coefficients of variation for each of our four imaging parameters to aid in the interpretation of our findings.

## 2 Methods

The research study was approved by the North West − Greater Manchester East Research Ethics Committee (Reference: 18/NW/0097), the Administration of Radioactive Substances Advisory Committee (ARSAC), and the University of Manchester research ethics committee. Written informed consent was obtained from all participants, who were recruited using the UK Join Dementia Research platform, Greater Manchester Mental Health NHS Foundation Trust memory clinic, word of mouth referrals or through advertisement at local University of the Third Age groups. Subjects aged between 60 – 80 years were recruited, including those with and without cardiovascular risk factors and with a range of cognitive complaints ranging from a diagnosis of mild cognitive impairment to normal cognition. Subjects were excluded from the study if they had depression (geriatric depression scale (GDS-15) score > 10), history of severe head trauma, brain tumour or brain infection, epilepsy, moderate stroke with modified Rankin scale (mRS) > 1, untreated vitamin B_12_ deficiency or thyroid disease, recurrent psychotic or neurodegenerative disorders, or were on medication for psychiatric disorders, benzodiazepines, high dose anti-depressants or anticonvulsants; history of drug abuse or alcohol consumption > 35 units per week; MRI contraindications (screening test, gadolinium allergy, renal insufficiency (estimated glomerular filtration rate, eGFR < 60)).

Vascular disease risk and cognitive impairment were assessed at screening interviews. Cardiovascular disease risk was parameterised using the QRisk2 screening tool (Hippisley-Cox, 2008). This algorithm predicts the likelihood of the participant sustaining a stroke or heart attack over the next ten years and uses information on age, gender, ethnicity, cholesterol levels, blood pressure and treatment, diabetes, chronic kidney disease, atrial fibrillation, rheumatoid arthritis, body mass index and smoking status. Blood pressure was also measured before the first scanning session. Cognitive parameters collected during the interview include years of education, and MoCA (Nasreddine, 2005) score. A lower boundary of 17 was used to filter out those with more severe cognitive impairment in order to limit the study to people in the earlier stages of dementia when the impact of cerebrovascular disease may be different from later stages. For example, it might be expected that reduced metabolic demand due to loss of neuronal density would impact CBF at later stages. A subset of patients were invited to a second visit in order to test the repeatability of the imaging measurements. Blood pressure was again measured before scanning and a shorter imaging protocol was used as described below.

### 2.1 Image acquisition

All acquisitions were performed on a hybrid 3 T PET-MRI scanner (Signa PET/MR, GE Healthcare, Milwaukee, WI) using a 32-channel head coil (Nova Medical, Wilmington, MA). QSM used a sagittal flow compensated 3D multi-echo gradient echo sequence, with parameters: 1 mm isotropic voxels, 208 x 208 x 150 mm FOV, 8 TEs from 3 ms to 16.5 ms, TR 22.7 ms, flip angle 15°, ASSET acceleration (factor = 2) in the phase encoding direction, and a bipolar echo collection scheme. ASL data was collected using the GE ‘enhanced ASL’ prototype with pseudo-continuous (pCASL) labelling, background suppression, vascular crushing, and a 3D spiral FSE read-out, with 6 spiral arms and 512 encoding points giving a resolution of 1.7 x 1.7 x 4 mm^3^. Image parameters were TE = 10.7 ms, TR = 5692 ms, 220 x 220 x 144 mm FOV. Two versions of the ‘3-delay’ sequence were applied, one with post-label delay times (PLDs) of (700, 1297 and 2158 ms) and label duration of (573, 885, 2024 ms), and a second with PLDs (1000, 1573 and 2024 ms) and label durations of (573, 885, 2024 ms), chosen in order to cover an equally distributed range of PLDs to allow robust estimation of both CBF and ATT. The corresponding label durations are set to approximately equalise the signal at each PLD; i.e. the longer PLDs have longer labelling durations. Prior to the ASL scan, a zero TE (ZTE) scan for PET attenuation correction was collected (3D center-out radial acquisition; voxel size 2.06 x 2.06 x 2.4 mm^3^; 116 slices of 2.4 mm thick, field of view (FOV) 26.4x26.4 cm^2^; flip angle 0.8˚; bandwidth ± 62.5 kHz; TR = 400 ms; TE = 0.016 ms; acquisition time 40 s). In addition, a proton density image with the same FOV was collected for use as the ‘M_0_′ image of equilibrium magnetisation, for calibration purposes. A 3D T1w magnetisation-prepared rapid gradient echo (MPRAGE) image and 3D T2w fluid attenuated inversion recovery (FLAIR) image were also collected with 1 mm isotropic resolution.

Individual haematocrit (HCT) was measured from a blood sample taken just prior to the scan. For accurate estimation of SaO_2_, the oxygen content of exhaled gas was monitored throughout the scan using a nasal cannula and an ADInstruments (Dunedin, New Zealand) gas analyser. The data was collected using LabChart and exported to Matlab.

The images described above were collected as part of a comprehensive simultaneous positron emission tomography-MR (PET-MR) examination in which a 185 MBq Bolus of [^18^F]flutemetamol was injected into an antecubital vein while the participants were inside the scanner at the start of the scan.

Following [^18^F]flutemetamol injection, the MRI scans were collected in the following order: ZTE, ASL, T1w MPRAGE, T2w FLAIR, QSM with a total scan duration of approximately 45 min. The participants were then removed from the scanner to rest in a quiet room before a second scanning session between 70 and 120 min post PET injection. The MR images collected in this second session of the first visit are not relevant to the work in this paper and will be reported elsewhere. PET data were acquired throughout both scanning sessions.

If subjects took part in a second visit, then a curtailed MR-only protocol list was used. ASL, T1w MPRAGE, and QSM sequences described above were repeated in the same order. HCT and oxygen content were resampled in the manner described above.

### 2.2 Image analysis

All image and oxygen trace manipulations were performed in Matlab (v2017a, The MathWorks, Inc., Natick, MA). Datasets with poor QSM image quality, such as excessive ringing, were excluded from the statistical analysis after a qualitative assessment.

#### 2.2.1 PET

PET images were reconstructed with full corrections including corrections for scatter and attenuation using similar parameters to those in the ADNI project,(Jagust, 2015) incorporating time-of-flight information (VPFX, 2.08 mm x 2.08 mm x 2.78 mm voxels, 4 iterations, 28 subsets, no resolution modelling, no post-filtering). An AC map was generated from ZTE MRI segmentation and used to correct the PET image using a post-processing pipeline provided by the manufacturer, based on the method proposed by Wiesinger et al (Wiesinger, 2016). The data was acquired in list mode and histogrammed into ten 5-minute frames from 70 to 120 min post injection. Four images between 90–110 min post-injection were co-registered and averaged to produce images of average radioactivity concentration which were assessed in terms of amyloid ‘positivity’ on a Yes/No basis by a neurologist with 30 years of experience of amyloid PET reporting (KH).

#### 2.2.2 T1w segmentation

T1w images were used to parcellate the brain into different regions of venous drainage from which CBF and ATT parameters can be extracted to match the veins identified on QSM. Before segmentation, bias field correction was applied using FSL(v6) (Jenkinson et al., 2012) “*FAST*”. Segmentation was performed with the Freesurfer (Fischl, 2012) v6.0.0 image analysis suite (http://surfer.nmr.mgh.harvard.edu/), using the Destrieux atlas (Destrieux et al., 2010), resulting in 180 cortical and sub-cortical regions from which the venous drainage territories can be delineated.

#### 2.2.4 SvO_2_ from quantitative susceptibility mapping data analysis

Estimates of SvO_2_ in major veins were extracted from the QSM images using the analysis methods described in previous work (McFadden, 2021). In brief, susceptibility maps were calculated from the real and imaginary QSM data using the *MEDI* + *0* algorithm (Liu, 2013, de Rochefort, 2010, Liu et al., 2018, Liu, 2012) (http://pre.weill.cornell.edu/mri/pages/qsm.html) for Matlab. Each acquired dataset was partitioned by echo polarity with odd or even echo datasets analysed separately and susceptibility maps averaged (McFadden, 2021). A copy of the final susceptibility map was created and registered to the segmented T1w image using FSL ‘*FLIRT’* algorithm. The outermost voxels at the edge of the brain were removed from the susceptibility map using a 3D single iteration erosion algorithm developed in-house in order to minimise tissue-air boundary errors resulting from imperfect background field removal.

Five venous structures of varying geometries, locations, and orientations were selected from an anatomical textbook (Rhoton, 2002) on the basis of the large size, prominence, regular anatomy, and drainage territory: SS − straight sinus, SSS V − superior sagittal sinus (vertical segment posterior to central sulcus), ICVs – internal cerebral veins, BVs – basal veins, SSS H – superior sagittal sinus (horizontal segment anterior to central sulcus). The territories associated with the Sylvian vein were not included due to the proximity of air-filled sinus producing susceptibility artefacts and consequentially unreliable estimates. The tentorial sinus regions were also excluded because of the highly variable anatomy yielding in some cases veins that were affected by partial volume effects or susceptibility artefacts. Regions containing each vein were initially manually identified using MRIcron v6.6 by drawing a rough outline on consecutive slices containing the vein, thereby creating a 3D volume. Each region of interest (ROI) was then refined by thresholding the susceptibility map, using a lower boundary of 130 ppb (parts per billion) and an upper boundary of 1000 ppb so that only venous voxels were included. Finally, the 90th percentile of the remaining non-zero voxels within each ROI was extracted.

The following relationship was used to derive SvO_2_ in each region (Fan, 2014):

$${\Delta \chi }_{\mathrm{vein}-CSF}=\left(1-{\mathrm{SvO}}_{2}\right)∙{\Delta \chi }_{0}∙Hct+\left({\Delta \chi }_{\mathrm{Hb}-H_{2}O}∙HCT\right)$$

where ${\Delta \chi }_{\mathrm{vein}-CSF}$ is the susceptibility difference between the vein and cerebrospinal fluid (CSF) in ppm, ${\Delta \chi }_{\mathrm{Hb}-H_{2}O}$ is the susceptibility difference between fully oxygenated red blood cells and water (−0.03 parts per million (ppm) in cgs units) (Weisskoff and Kiihne, 1992), Hct is the haematocrit, and ${\Delta \chi }_{0}$ is the susceptibility difference per unit haematocrit between fully oxygenated and fully deoxygenated red blood cells (0.27 ppm in cgs units) (Spees et al., 2001).

### 2.3 SaO_2_ from oxygen trace data analysis

The exhaled oxygen trace was exported to Matlab and the end-tidal oxygen percentages during the QSM acquisition were estimated from the peaks using the *findpeaks* function. The median end-tidal value was converted to SaO_2_ using the Severinghaus equation (Severinghaus, 1979):

$${\mathrm{SaO}}_{2}\left(\text{\%}\right)=\frac{1}{1+\left(\frac{23400}{{\mathrm{EtO}}_{2}^{3}+150∙{\mathrm{EtO}}_{2}}\right)}$$

### 2.4 CBF, ATT & CMRO_2_ from ASL

CBF maps were calculated in Matlab following recommendations of the white paper (Alsop, 2015) using a single blood compartment model (Parkes and Tofts, 2002) to fit on a voxel-wise basis for CBF and ATT. Data from both of the ASL acquisitions (each with 3 PLDs) were modelled together using one kinetic model. Fixed parameters: labelling efficiency = 0.85; blood–brain partition coefficient = 0.9. Voxel-wise M_0_ (equilibrium magnetisation) calibration was used. Arterial blood T_1_ estimates were calculated on an individual basis using the HCT from the blood sample taken prior to the scan and using the following equation (Lu et al., 2004):

$$\frac{1}{T_{1}}=0.52∙HCT+0.38$$

**Fig. 1:**
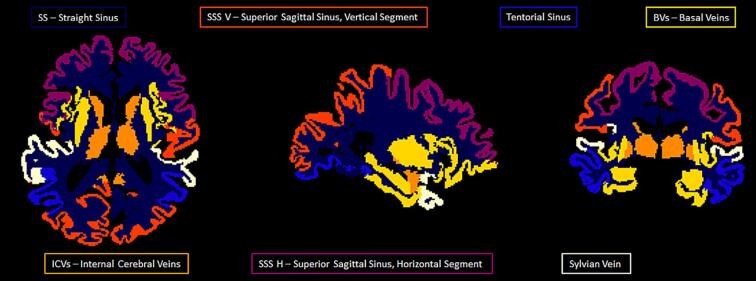
Axial, sagittal, and coronal views of Freesurfer regions drained exclusively by selected veins. This shows the exclusive territory drained by each vein with the total territory for each vein as follows: SSS V_total_ = SSS V + SSS H; SS_total_ = SS + ICVs + BVs. The region drained by the Sylvian veins was excluded as OEF values could not be reliably estimated from it due to strong susceptibility-gradient induced artefacts and conservative brain masking. The region drained by the tentorial sinuses was excluded due to the variable anatomy.

The median CBF and ATT values were extracted from the vascular territory of the selected veins. This was achieved through co-registration of the CBF maps with the T1w images (using the ASL calibration images), segmented according to the Destrieux atlas. The segmented regions were allocated to a vein if drained by it (Fig. 1) using a textbook as a reference (Rhoton, 2002). Note that Fig. 1 shows the regions drained exclusively by each vein; however because some of the identified smaller veins flow directly into larger veins, the oxygen saturation derived from these larger veins are likely to be a composite of both exclusive and upstream territories. To harmonise the regional specificity of our OEF and CBF values, the CBF values assigned to each vein were calculated using both exclusive and upstream territories. Specifically, the SS CBF value was calculated from the values obtained in the ICVs, BVs, and SS-exclusive territories and the SSS V CBF value was calculated from the values obtained in the SSS V and SSS H exclusive territories.

Finally, CMRO_2_ was calculated for each region using Eq. (2), where [O_2_]_a_ in μmol O_2_/100 ml blood was calculated using (Xu et al., 2009):

$${\left[O_{2}\right]}_{a}=\left[Hb\right]∙C_{a,Hb}$$

where [Hb] in g/100 ml blood is the concentration of haemoglobin and C_a,Hb_ is the oxygen carrying capacity of a haemoglobin molecule (55.6 μmol O2/g). [Hb] is estimated as HCT/3 (Doig and Zhang, 2017).

### 2.5 Statistical analysis

Statistical analyses were conducted using R 4.0.3 (R Core Team, 2020).

To determine how each of the four imaging parameters relate to vascular disease, four independent linear mixed models were constructed using the *lmerTest* package, with: OEF, CBF, CMRO_2_, or ATT analysed separately as the dependent variable; QRisk score and region as additional covariates and fixed effects; and subject as a random effect. Age was not added as fixed effect as age is used to calculate QRisk score and therefore would have been collinear. Subject is included as there is likely to be a global difference in imaging parameters between people. Interaction between QRisk and region was included to determine if any relationships were regionally-dependent. The model was analysed using type III analysis of variance (ANOVA).

$$\mathrm{MRI}\;\mathrm{variable}∼\;QRisk*Region+\left(1|Subject\right)$$

To investigate if OEF was increasing to compensate for reduced CBF and whether this was associated with prolonged ATT, the relationship between OEF and CBF or ATT was also tested using a linear mixed model which was constructed with OEF as the dependent variable; CBF or ATT, region and their interaction as fixed effects; with age as a covariate and subject as a random effect. The model was analysed using the type III ANOVA.

$$\mathrm{OEF}∼\;CBF\;or\;ATT*Region+\left(1|Subject\right)$$

Finally, we evaluated if our imaging parameters were associated with cognitive impairment. The first analysis concerning associations between vascular imaging parameters and QRisk did not reveal any regionally-specific effects and so, for this subsequent analysis, we used a single, global metric for each participant and variable. For OEF, we chose the estimate from the SSS V, as this drains much of the grey matter and is the region of interest most often used for global methods of estimating OEF. For CBF and ATT we used the average value from the whole of the cortical grey matter. Global CMRO_2_ was calculated using these estimates and equation (Dichgans and Leys, 2017). Linear models were constructed with MoCA score as the dependent variable, and separately with OEF, CBF, CMRO_2_, or ATT as a covariate and amyloid status as a fixed effect. This model was analysed with a type II ANOVA.

MoCA∼MRIvariable+AmyloidStatus

#### 2.5.1 Inter-session repeatability

For each of the imaging parameters, the within-subject standard deviation $\sigma_{\mathrm{inter}}$ was calculated for each vein and associated draining territory using the Bland-Altman formula (Bland and Altman, 1996):

$$\sigma_{\mathrm{inter}}=\sqrt{\frac{\sum_{i=1}^{n}{\left(x_{1i}-x_{2i}\right)}^{2}}{2n}}$$

where n is the number of subjects undergoing both scanning session, and $x_{1i}$ and $x_{2i}$ are the imaging estimates for each subject i at the 1st and 2nd scan respectively. These were converted to inter-session within-subject coefficients of variation (CoV) for each vein by dividing this value by the mean value across the n participants.

## 3 Results

Twenty-nine people met the inclusion/exclusion criteria and were scanned. The imaging data were discarded for four of these subjects because of poor image quality in the QSM images, and for one due to a failure of PET tracer delivery to the scanning site. The remaining 24 volunteers (age 69.6 ± 5.3 years; 14 females) had a range of vascular risk factors (QRisk 18.7 ± 10.8 %, range 5.1 – 45.4; Montreal cognitive assessment (Nasreddine, 2005), MoCA scores 26.7 ± 3.4, range 17 – 30; geriatric depression score GDS 1.1 ± 1.4 and Years of Education 16.7 ± 4.6. Seven of the participants were amyloid positive, 17 amyloid negative.

**Fig. 2:**
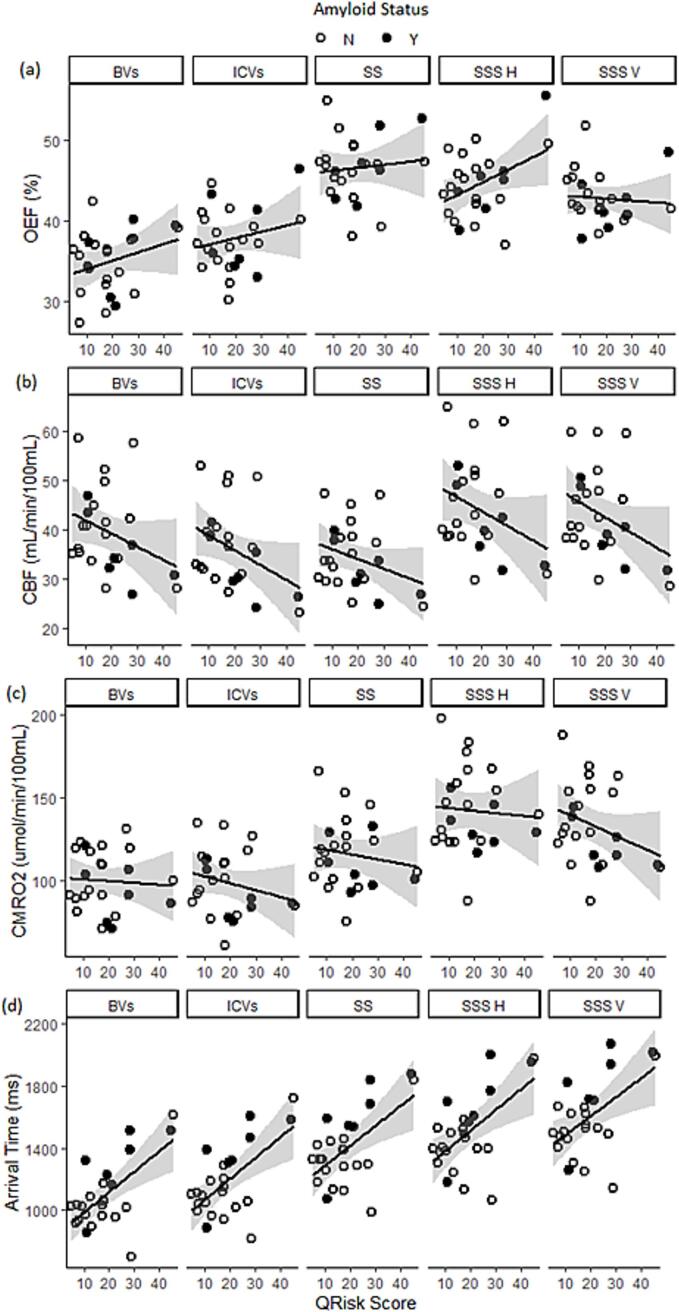
Relationship between regional vascular imaging measurements and vascular disease risk. regional vascular imaging measurements of a)oxygen extraction fraction (OEF), b) cerebral blood flow (CBF), c) cerebral metabolic rate for oxygen (CMRO_2_) and d) arterial transit time (ATT) with QRisk, a measure describing the likelihood of the participant sustaining a stroke or heart attack over the next ten years. Dark circles represent people with amyloid positive status (Y) as determined from amyloid PET imaging and open circles people who are amyloid negative (N). BVs – basal veins; ICVs – internal cerebral veins; SS – straight sinus; SSS H – superior sagittal sinus (horizontal segment); SSS V – superior sagittal sinus (vertical segment). Shaded regions are confidence intervals calculated along with the linear fit using the geom_smooth function in R.

A strong positive correlation between QRisk score and ATT (β = +13.2, *p* = 0.002) was observed (Table 1 and Fig. 2). The regression coefficients β are the predicted change in the imaging parameter per point increase of the driving variable (QRisk); i.e there is a 132 ms increase per 10 point increase in QRisk. Neither CBF, OEF nor CMRO_2_ showed a significant relationship with QRisk. However, a trend was observed for CBF (β = -0.3, *p* = 0.094). There were significant regional differences in all four vascular imaging parameters, but no regional differences in their associations with QRisk.

**Table 1:** Analysis of variance tables examining the association between vascular imaging parameters, vascular disease risk (QRisk).

		Df	SS	MS	F value	p value
OEF	Qrisk Score	1	11.8	11.8	1.6	0.225
	**Region**	**4**	**641.2**	**160.3**	**21.2**	**<0.001**
	Qrisk:Region	4	51.3	12.8	1.7	0.157
	Residual Variance	7.5				
CBF	*Qrisk Score*	*1*	*9.6*	*9.6*	*3.1*	*0.094*
	**Region**	**4**	**555.1**	**138.8**	**44.3**	**<0.001**
	Qrisk:Region	4	22.8	5.7	1.8	0.132
	Residual Variance	3.1				
CMRO_2_	Qrisk Score	1	65.6	65.6	0.7	0.399
	**Region**	**4**	**10195.6**	**2548.9**	**28.8**	**<0.001**
	Qrisk:Region	4	593.4	148.4	1.7	0.163
	Residual Variance	88.6				
ATT	**Qrisk Score**	**1**	**18446.0**	**18446.0**	**12.0**	**0.002**
	**Region**	**4**	**1059733.0**	**264933.0**	**172.1**	**<0.0001**
	Qrisk:Region	4	2860.0	715.0	0.5	0.762
	Residual Variance	1540.0				

Bold denotes a significant result, italics a trend.

Df (degrees of freedom), SS (sum of squares), MS (mean squares).

**Fig. 3:**
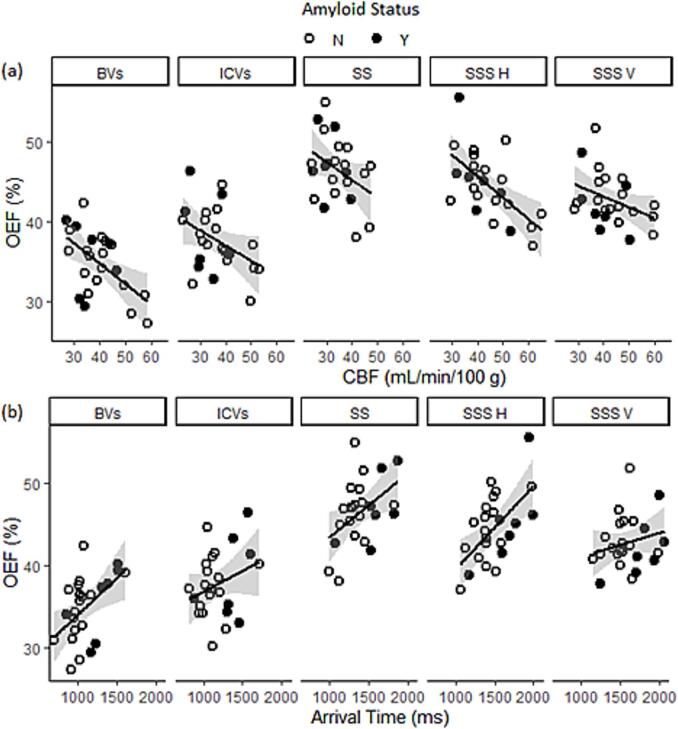
Compensatory relationship between OEF and CBF or ATT. Regional vascular imaging measurements of oxygen extraction fraction (OEF) against a) cerebral blood flow (CBF) and b) arterial transit time (ATT). Dark circles represent people with amyloid positive status (Y) as determined from amyloid PET imaging and open circles people who are amyloid negative (N). BVs – basal veins; ICVs – internal cerebral veins; SS – straight sinus; SSS H – superior sagittal sinus (horizontal segment); SSS V – superior sagittal sinus (vertical segment). Shaded regions are confidence intervals calculated along with the linear fit using the geom_smooth function in R.

OEF had a significant negative correlation with CBF (β = -0.2, *p* = 0.002, Table 2 and Fig. 3a) and a significant positive correlation with ATT (β = 0.01, *p* = 0.003, Table 2 and Fig. 3b). Region accounted for significant variance for the CBF model (*p* = 0.005) and the ATT model (*p* = 0.039).

**Table 2:** Analysis of variance tables examining the association between OEF and CBF/ATT.

	Df	SS	MS	F value	p value
**CBF**	**1**	**93.8**	**93.8**	**11.9**	**0.002**
**Region**	**4**	**126.6**	**31.7**	**4**	**0.005**
CBF:Region	4	24.2	6	0.8	0.547
Residual Variance	7.9				
**ATT**	**1**	**80.5**	**80.5**	**10.7**	**0.003**
**Region**	**4**	**79.1**	**19.8**	**2.6**	**0.039**
ATT:Region	4	46.1	11.5	1.5	0.199
Residual Variance	7.5				

Bold denotes a significant result, italics a trend.

Df (degrees of freedom), SS (sum of squares), MS (mean squares).

**Fig. 4:**
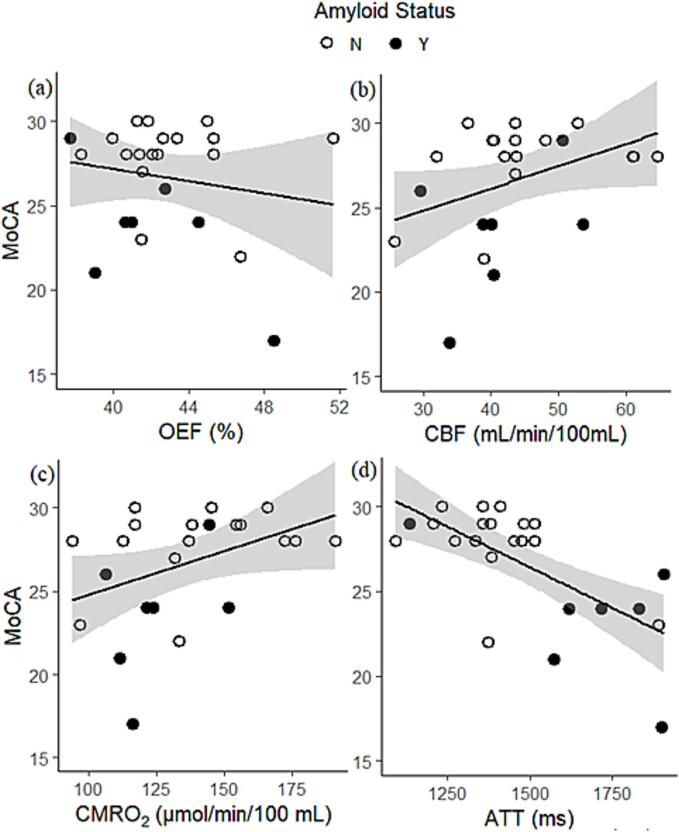
Relationship between vascular imaging measurements and MoCA score. Vascular imaging measurements of a) oxygen extraction fraction (OEF), b) cerebral blood flow (CBF), c) cerebral metabolic rate for oxygen (CMRO_2_) and d) arterial transit time (ATT) with MoCA, a global measure describing the degree of cognitive impairment for an individual. Dark circles represent people with amyloid positive status (Y) as determined from amyloid PET imaging and open circles people who are amyloid negative (N). Shaded regions are confidence intervals calculated along with the linear fit using the geom_smooth function in R.

Our cognition models revealed a significant association where an increase in ATT (β = -0.007, *p* = 0.01) was associated with a decrease in MoCA (Table 3 and Fig. 4). This equates to a 725 ms prolongation in ATT for a 5 point reduction in MoCA score. Amyloid status was also significantly associated with MoCA (Table 3) for all but the ATT model, where a trend was observed instead. Being amyloid positive resulted in a reduction in MoCA of between 3.9 and 4.6 for the models where the association was significant.

**Table 3:** Analysis of variance tables examining the association between vascular imaging parameters and cognition (MoCA score).

	Df	SS	F value	p value
OEF	1	17.5	2.5	0.129
**Amyloid Status**	**1**	**104.5**	**14.9**	**<0.001**
Residuals	21	147.2		
CBF	1	19.6	2.8	0.107
**Amyloid Status**	**1**	**74.9**	**10.8**	**0.003**
Residuals	21	145.1		
CMRO_2_	1	14.7	2.1	0.166
**Amyloid Status**	**1**	**68.8**	**9.6**	**0.005**
Residuals	21	150		
**ATT**	**1**	**43.4**	**7.5**	**0.012**
Amyloid Status	1	21.4	3.7	*0.068*
Residuals	21	121.3

Bland-Altman analysis (Table 4) yielded inter-session repeatability CoVs of 3.6 % to 8.1 % for OEF; 7.9 % to 14.1 % for CBF; 5.1 % to 7.1 % for ATT; and 7.7 % to 16.7 % for CMRO_2_, depending on region.

**Table 4:** Regional mean values and coefficient of variation of each vascular imaging parameters.

Estimate	Statistic	SSS V	SSS H	SS	BVs	ICVs
OEF	Mean (%)	42.7	44.5	46.5	34.9	37.7
	S. Error	0.7	0.9	0.8	0.8	0.8
	CoV (%)	3.6	4.9	6.3	5.8	8.1
CBF	Mean (ml/100 ml/min)	43.0	44.2	34.4	39.6	36.2
	S. Error	1.9	2.0	1.4	1.8	1.7
	CoV (%)	13.0	14.1	7.9	10.6	10.3
CMRO_2_	Mean (umol/100 ml/min)	133.0	141.9	115.6	99.4	98.4
	S. Error	5.0	5.1	4.3	3.7	4.0
	CoV (%)	13.4	16.7	12.4	10.6	7.7
ATT	Mean (ms)	1592	1497	1399	1102	1183
	S. Error	50.1	50.5	48.6	46.0	47.6
	CoV (%)	5.1	7.1	7.1	6.7	6.4

From left to right: SSS V − superior sagittal sinus (vertical segment), SSS H – superior sagittal sinus (horizontal segment), SS − straight sinus, BVs – basal veins, ICVs – internal cerebral veins.

## 4 Discussion

In this study, we evaluate the impact of subclinical cerebrovascular disease as parameterised by QRisk score on CBF, OEF, CMRO_2_, and ATT, on a regional basis. We also evaluate the association of CBF and ATT with OEF to investigate the compensatory relationship between them. We found evidence that ATT was significantly altered in those with greater vascular risk and in those who were cognitively impaired, and that OEF was significantly increased in those with elevated ATT or CBF.

ATT rose significantly by 132 ms per 10 point increase in QRisk, a ∼ 10 % increase, believed to be due to the expected increase in cerebrovascular resistance as structural alterations to the microvascular network occur in association with elevated QRisk, as there is evidence that capillaries, should they be regenerated after death or damage, have increased tortuosity (Buée, 1994). This increase is comparable to the difference in ATT between the younger and older participants reported elsewhere (Juttukonda, 2021). Further work is necessary to delineate the contributions of individual vascular risk factors and aging, which this study is underpowered to do. These findings support the use of these vascular imaging parameters as early markers of cerebrovascular disease. There were regional differences for all measures, but no regional interactions, suggesting that baseline regional heterogeneity in the vascular measures is not associated with greater or lesser degrees of regional compensation. While the association between ATT and QRisk was significant, the expected relationship between QRisk and CBF was not significant, likely due to both the smaller effect size and the larger CoV of the CBF metric (Table 4) which may reflect greater physiological stability of the ATT measure over CBF. The lack of a significant CBF-QRisk relationship does not match expectations as CBF is known to be reduced with both hypertension (Barry, 1985) and aging (Parkes et al., 2004), both of which are input variables into QRisk. However, there is a trend towards this relationship which perhaps would prove significant in a larger sample size, counteracting the high variability in the CBF measurement (reflected in the somewhat poor repeatability).

The lack of association between QRisk and CMRO_2_ (Fig. 2c) suggests that OEF elevation could be occurring in compensation for reduced CBF, thereby providing the required oxygen, and consequentially mitigating the effect of reduced blood supply on cognition. In support of this, we observe a significant association between CBF and OEF across all regions (Fig. 3a). The varying intercepts of this relationship reflect the regional differences of these measures, with the regional variability in OEF being similar to that observed using TRUST (Jiang, 2023). Longer ATT is also associated with elevated OEF and it is plausible that lengthened microvascular networks and/or longer transit times are enabling increased OEF as originally described by Krogh and Erlang (Krogh, 1919). This resonates with recent longitudinal (Lin, 2023) and cross-sectional (Kang, 2022) studies where OEF was observed to rise in those with a greater number of vascular risk factors and with faster growing volumes of white matter hyperintensities. The cross-sectional study also demonstrates elevated OEF in associated with lower CBF and no association between CBF and WMH volume, which supports the potential utility of OEF in small vessel disease. On the other hand, recent work (Le, 2023) has demonstrated OEF-CBF association in younger individuals who were unlikely to have the potentially lengthened and rarefied capillary network of an older individual, suggesting that the compensatory mechanism might also occur during dynamic processes such as vessel tone modulation.

Our MoCA findings offer an insight into the degree to which microvascular alterations and OEF compensation are associated with cognitive impairment. We found that prolonged ATT was significantly associated with poorer cognition, suggesting that the degree of vascular damage is affecting cognition (Fig. 4, Table 3). CMRO_2_ however, did not decline with QRisk and was not directly associated with MoCA, contrary to our hypothesis. This may be because we are measuring CMRO_2_ at rest, when the deficiencies in oxygen supply are not apparent and metabolic demand can be met. It may also be attributable to the fact that CMRO_2_ also has relatively poor repeatability. The lack of a relationship between cognition and imaging measures other than ATT does however align with another study (Catchlove, 2018) which also found no relationships between cognition and either venous oxygen saturation or CMRO_2_. That the presence of amyloid would be associated with impaired cognition was expected but, because of the relatively few positive cases in our dataset, we were unable to fully explore the degree to which this impairment was independent of that associated with ATT.

The mean values we obtained for our imaging parameters (Table 4) were in general agreement with the literature (Bulte, 2012, Ito, 2004, Ibaraki, 2010, Bremmer, 2011, Gauthier and Hoge, 2012, Federau, 2017). The fact that our CBF values were at the lower range is likely due to the age of our cohort. While all vascular metrics showed significant regional variation (Table 1), the association with Qrisk was not regionally dependent. For OEF, regional differences may be due to the varying size and orientations of the veins used in this study. While we took steps to mitigate the impact of partial voluming, it’s influence cannot be fully ruled out in the case of the smaller vessels such as the BV and ICV whose lower OEF values would be consistent with partial voluming. The regional differences in CBF are to expected due to the differing grey/white matter composition of the vascular territories. For example, the SS region contains proportionately more white matter than other regions and hence has lower CBF. Similarly, ATT differences are due to known differences in arterial path length of the differing regions; for example, the SSSV region containing ‘watershed’ regions with proportionately longer path lengths, and consequently longer ATT, than other regions. The lack of regional differences in the association between the vascular metrics and Qrisk, or indeed between CBF and OEF could indicate both that the origin of vascular dysfunction is global and that none of our brain regions were uniquely vulnerable to the effects.

There are several limitations to our study, of which a small number of participants is the most important. Our brain regions of interest, while an improvement over a global measure, are still quite large. The absence of true regional specificity of the larger veins are also disadvantageous. Using a higher resolution and/or greater field strength would have increased the number of reliable small veins and thereby improved the regional specificity of the OEF and consequently CMRO_2_ estimates. However, the acquisition times risked becoming unfeasibly long with higher resolution scans. One further concern is the accuracy of the regional CMRO_2_ given the assumption that the large draining veins are representative of OEF within the tissue that the vein is draining. The repeatability of our vascular imaging measurements are, however, reasonably good and commensurate with other studies (Liu et al., 2013, Bremmer, 2011, Lajoie et al., 2016, Coles, 2006, Chen et al., 2011) except for CBF which is somewhat poor, though not uncommonly so, compared to the literature (Liu et al., 2013, Bremmer, 2011, Lajoie et al., 2016, Coles, 2006, Chen et al., 2011, Merola et al., 2018, Salluzzi et al., 2022, Wu et al., 2014).

In conclusion, this work shows that multi-delay time ASL and QSM can provide MRI measurements of regional CBF, ATT, OEF and CMRO_2_ that could be useful to detect vascular changes associated with cognitive impairment. We demonstrated that those most affected by vascular dysfunction had both the highest level of oxygen extraction but also the greatest amount of cognitive impairment. In particular we have demonstrated the potential utility of ATT as a tool sensitive to both vascular dysfunction and mild cognitive impairment.

## CRediT authorship contribution statement

**John McFadden:** Writing – review & editing, Writing – original draft, Visualization, Software, Methodology, Investigation, Formal analysis, Data curation, Conceptualization. **Julian Matthews:** Writing – review & editing, Supervision, Project administration, Methodology, Formal analysis, Conceptualization. **Lauren Scott:** Writing – review & editing, Formal analysis. **Karl Herholz:** Writing – review & editing, Validation, Investigation. **Ben Dickie:** Writing – review & editing, Supervision, Methodology, Investigation, Formal analysis, Data curation. **Hamied Haroon:** Writing – review & editing, Methodology, Formal analysis. **Oliver Sparasci:** Project administration, Methodology. **Saadat Ahmed:** Methodology. **Natalia Kyrtata:** Methodology. **Geoffrey J.M. Parker:** Writing – review & editing, Supervision, Project administration, Funding acquisition, Conceptualization. **Hedley C.A. Emsley:** Methodology. **Joel Handley:** Methodology. **Maélène Lohezic:** Writing – review & editing, Supervision, Software, Resources, Project administration, Methodology. **Laura M. Parkes:** Writing – review & editing, Validation, Supervision, Project administration, Methodology, Investigation, Funding acquisition, Formal analysis, Data curation, Conceptualization.

## Declaration of Competing Interest

The authors declare that they have no known competing financial interests or personal relationships that could have appeared to influence the work reported in this paper.

## Data Availability

The authors do not have permission to share data.
